# Fouling process of hemodiafiltration membranes by hemodialysis and hemodiafiltration therapy

**DOI:** 10.1007/s10047-025-01497-9

**Published:** 2025-02-27

**Authors:** Yoshihiro Tange, Masashi Kawakami, Shingo Takesawa

**Affiliations:** 1 Department of Advanced Medical Sciences, Faculty of Medicine, Oita University, 1-1 Idaigaoka, Hasama-Machi, Yufu, Oita 879-5593 Japan; 2 Graduate School of Health Sciences, Kyushu University of Medical Science, 1714-1 Yoshinomachi, Nobeoka, Miyazaki 882-8508 Japan; 3 Department of Clinical Engineering, Tokatsu Clinic Hospital, 865-2 Hinokuchi, Matsudo, Chiba 271-0067 Japan

**Keywords:** Intermittent infusion hemodiafiltration, Online hemodiafiltration, Fouling

## Abstract

During hemodialysis, proteins, such as albumin and globulin, are deposited on the dialysis membrane surface, causing fouling that affects solute removal and biocompatibility. This study aimed to measure the filtration coefficient as an index of dialysis membrane conditions in hemodialysis, pre-dilution online hemodiafiltration, and intermittent infusion hemodiafiltration modes using two different hemodiafiltration membranes ex vivo. The filtration coefficients of hemodiafiltration membranes in hemodialysis, pre-dilution online hemodiafiltration, and intermittent infusion hemodiafiltration modes were continuously measured for 123 min using 2 L bovine blood, which was adjusted with 32% hematocrit and 6.5 g/dL of total proteins. Polysulfone and cellulose triacetate were used as test membrane materials, and both membrane structures were asymmetric. The first fouling step was observed 20 s after filtration of both polysulfone and cellulose triacetate membranes in each mode. Thereafter, the filtration coefficient recovered in the pre-dilution online hemodiafiltration mode. However, it plateaued in the cellulose triacetate membrane and decreased in the polysulfone membrane. A flushing effect of the intermittent infusion hemodiafiltration mode was observed in both the cellulose triacetate and polysulfone membranes. The differences in fouling steps in each of the three modes—hemodialysis, pre-dilution online hemodiafiltration, and intermittent infusion hemodiafiltration—can be identified by continuously measuring filtration coefficient values.

## Introduction

In 2010, 2.6 million individuals underwent renal replacement therapy (RRT) worldwide, which is expected to double by 2030 [1]. A survey in Japan reported that 339,841 patients underwent RRT in 2018. Among them, 121,634 patients were on hemodiafiltration (HDF) at the end of 2018, of whom 86,231 (70.9%) and 31,681 (26.0%) had been on online HDF and intermittent infusion HDF (I-HDF), respectively [2].

Online HDF, which utilizes an ultrapure substitute dialysis fluid and is directly injected into the blood of the patient, reduces patient mortality [3, 4] and lowers cardiovascular event occurrences [5], making it the standard treatment for patients receiving RRT. Recently, I-HDF using back filtration with ultrapure dialysis fluid was developed [6]. In I-HDF, ultrapure dialysis fluid is infused via an HDF membrane using intermittent back filtration to increase the circulating blood volume of the patient during dialysis therapy. A clinical trial showed that the removal rates of low-molecular-weight and medium-to-high-molecular-weight substances are significantly lower and higher, respectively, with I-HDF than with conventional hemodialysis (HD). I-HDF also results in significantly less albumin leakage than HD [7].

In contrast, online pre-dilution HDF demonstrates a significantly higher removal rate of medium- and large-molecular-weight solutes and higher albumin leakage than I-HDF [8]. Thus, I-HDF may be a suitable RRT method for patients with malnutrition and advanced age. In addition, in in vitro evaluation, I-HDF showed solute clearance recovery by back filtration, which is a characteristic I-HDF feature [9]. However, the fouling process in I-HDF is not fully understood because intermittent filtration from the blood side to the dialysate side and vice-versa is repeated.

Therefore, we aimed to conduct HD, pre-dilution online HDF, and I-HDF to clarify the membrane fouling characteristics using two different HDF membranes ex vivo.

## Materials and methods

### Bovine blood

Bovine blood samples were obtained from a local distributor (Tokyo Shibaura-Zoki, Tokyo, Japan). We adjusted it to attain a hematocrit of 32% and 6.5 g/dL of total protein concentration using saline (Otsuka, Tokyo, Japan), plasma, and whole blood. The blood contained 20 mM of sodium citrate, which was used as an anticoagulant.

### HDF membranes

Polysulfone (PS) (ABH-22PA; Asahi-Kasei, Tokyo, Japan) and cellulose triacetate (CTA) (FIX-210Seco; NIPRO, Osaka, Japan) membranes were used. The specifications of both membranes are listed in Table 1. The number of fibers was counted individually.

**Table 1:** Hemodiafiltration membrane specifications

Manufacturer	Asahi-Kasei Co. Ltd	NIPRO Co. Ltd
Product name	ABH-22PA	FIX-210Seco
Hollow fiber material	PS	CTA
Membrane structure	Asymmetric	Asymmetric
Surface area (m^2^)	2.2	2.1
Inner diameter (μm)	200	200
Thickness (μm)	43	25
Length (mm)	266	254
Fiber length (mm)	288	283
Number of fibers	13,170	13,170
PV (mL)	131	125
Filling rate (%)	60	60
UFR (mL mmHg^−1^ h^−1^)	108	81
PVP	+	−

*PS* polysulfone, *CTA* cellulose triacetate, *PV* priming volume, *UFR* ultrafiltration rate, *PVP* polyvinylpyrrolidone

### Experiments

Using 2 L of bovine blood, we conducted HD, pre-dilution online HDF, and I-HDF experiments for 123 min. The blood (Q_B_) and dialysate (Q_D_) flow rates were set at 200 mL/min and 500 mL/min, respectively. The bovine blood temperature was set to 37 °C using a hot stirrer. Heparin (NP; NIPRO) was continuously infused at 3,000 U/h. Filtration was performed after circulating the blood flow pump for 1 min, and the pressures of the blood inlet (P_Bi_), blood outlet (P_Bo_), dialysate inlet (P_Di_), and dialysate outlet (P_Do_) sides of the HDF membrane were continuously recorded. A personal dialysis machine (DBG-03; Nikkiso, Tokyo, Japan) and dialysis fluid concentrate (AF-2; FUSO, Kawasaki, Japan) were used.

### HD mode

**Fig. 1:**
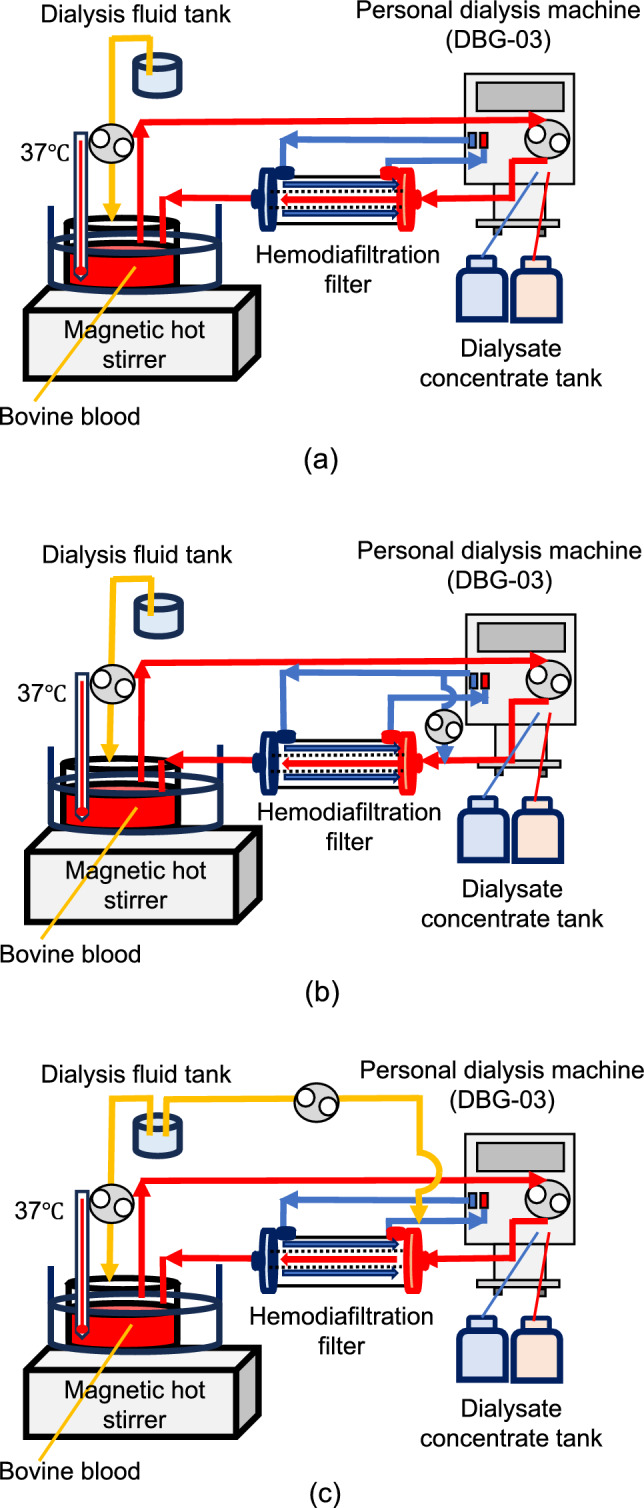
Schematic diagram of the experiments. Experiments are conducted ex vivo using 2 L of bovine blood at 37 °C. **a** Hemodialysis (HD) mode, **b** pre-dilution online hemodiafiltration (HDF) mode, and **c** intermittent infusion HDF (I-HDF) mode

The Q_B_, Q_D_, and ultrafiltration flow rate (Q_F_) were set to 200, 500, and 30 mL/min, respectively. The replacement fluid was delivered from the dialysis fluid tank at the same flow rate as the Q_F_ to the bovine blood. The total filtration volume was 3.69 L (Fig. 1a).

### Pre-dilution online HDF mode

The Q_B_, Q_D_, Q_F_, and supplemental flow rate (Q_S_) were set at 200, 500, 30, and 208 mL/min, respectively. The same volume flow rate of Q_F_ was added from the dialysis fluid tank to the bovine blood (Fig. 1b).

### I-HDF mode

The Q_B_, Q_D_, and Q_F_ were set to 200, 500, and 30 mL/min, respectively. Supplemental fluid (24 mL/min) was added to the dialysis tanks. The back filtration flow was caused by the dialysis fluid tank, and back filtration was injected at 200 mL/min for 20 s at 10 min intervals (Fig. 1c).

### ***Measurement of transmembrane pressure (TMP) and pressure differences in the inlet portion (ΔP***_***A***_***), outlet portion (ΔP***_***V***_***), and filtration coefficient (Lp)***

A pressure gauge was created using an analog-to-digital converter, pressure transducer, and direct current adapter, and it was measured at 5 s intervals. Pressure was measured continuously at four points: P_Bi_, P_Bo_, P_Di_, and P_Do_ of the HDF module. The pressure differences between the ΔP_A_ of the module, ΔP_V_, TMP, and Lp were calculated as the average values over 5 s using the following formula:

$${\Delta P}_{A}= {P}_{Bi}-{P}_{Do}$$

$${\Delta P}_{V} = {P}_{Bo}-{P}_{Di}$$

$$TMP =\frac{\left({P}_{Bi}+{P}_{Bo}\right)}{2}-\frac{\left({P}_{Di}+{P}_{Do}\right)}{2}$$

$${L}_{p}=\frac{{Q}_{F}}{TMP\times A}$$

where ΔP_A_ denotes pressure differences between the inlet portion (mmHg), ΔP_V_ denotes pressure differences between the outlet portion (mmHg), Lp denotes the filtration coefficient (mL m^−2^ h^−1^ mmHg^−1^), TMP denotes transmembrane pressure (mmHg), Q_F_ denotes the filtration flow rate (mL/h), and A denotes the membrane surface area (m^2^).

The initial fouling characteristics were obtained using the Lp, which was expressed on a logarithmic scale.

### Ethical approval

This was not required for this study.

## Results

**Fig. 2:**
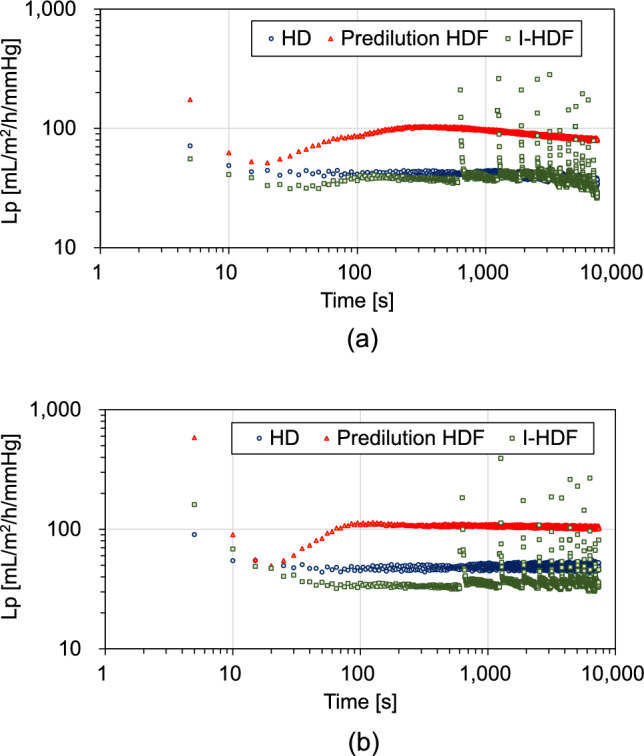
Changes in filtration coefficient (Lp) in the polysulfone (PS) and cellulose triacetate (CTA) membranes. **a** PS and **b** CTA membranes. In each experimental mode of PS and CTA, the Lp decreases 20 s after the start of filtration in a two-step process in HD and I-HDF and three-step in pre-dilution online HDF modes in both membranes

Figure 2 shows the Lp changes in the HD, pre-dilution online HDF, and I-HDF modes during the experiment in the PS (Fig. 2a) and CTA (Fig. 2b) membranes. In each experimental mode, the Lp decreased 20 s after the start of filtration. During HD, the Lp decreased in both membranes (Fig. 2). With pre-dilution online HDF, the Lp decreased rapidly, recovered and decreased in the PS membrane (Fig. 2a), and was maintained in the CTA (Fig. 2b). The I-HDF mode also decreased the Lp in both membranes, and the Lp recovered temporally with intermittent infusion.

**Fig. 3:**
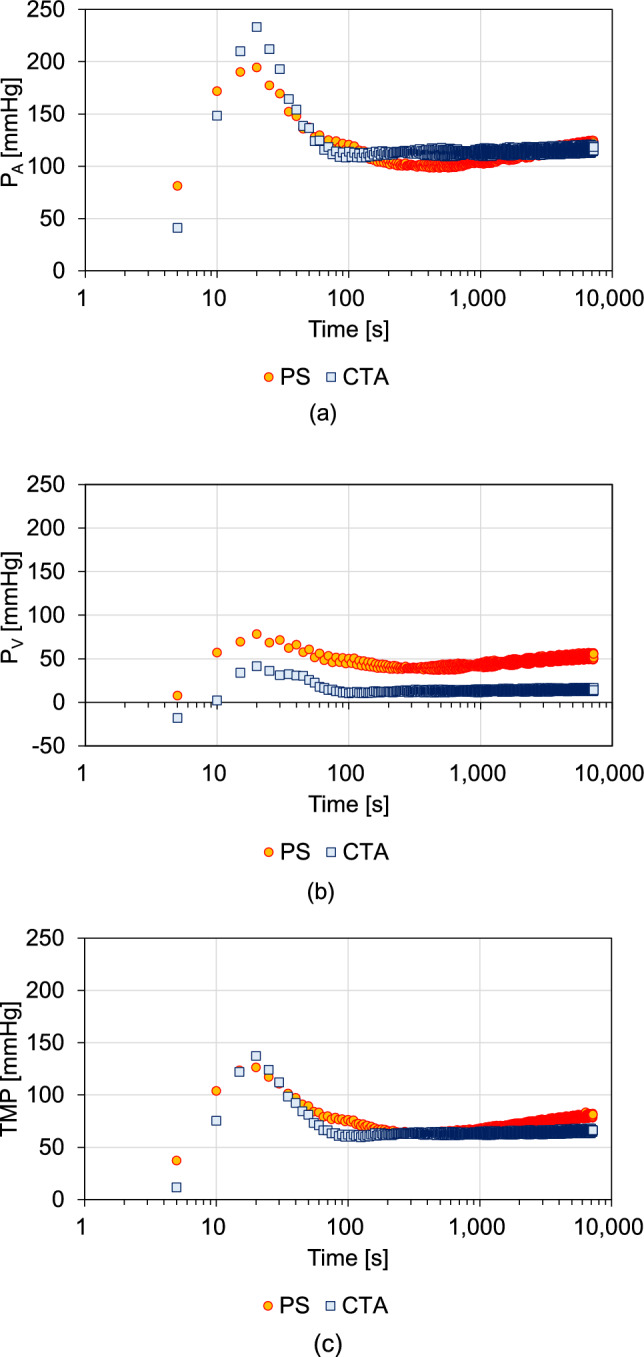
Changes in the pressure differences between the inlet portion (ΔP_A_) of hemodiafiltration membranes, outlet portion (ΔP_V_), transmembrane pressure (TMP), and Lp in the pre-dilution online HDF mode. The pressures in the pre-dilution online hemodiafiltration mode are shown for **a** ΔP_A_, **b** ΔP_V_, and **c** TMP

Figure 3 shows the ΔP_A_, ΔP_V_, and TMP changes in the pre-dilution online HDF mode. ΔP_A_ (Fig. 3a), ΔP_V_ (Fig. 3b), and TMP (Fig. 3c) rapidly increased 20 s after the start of filtration and then decreased over the next 80 s, plateauing in the CTA and slightly increasing in the PS membranes.

**Fig. 4:**
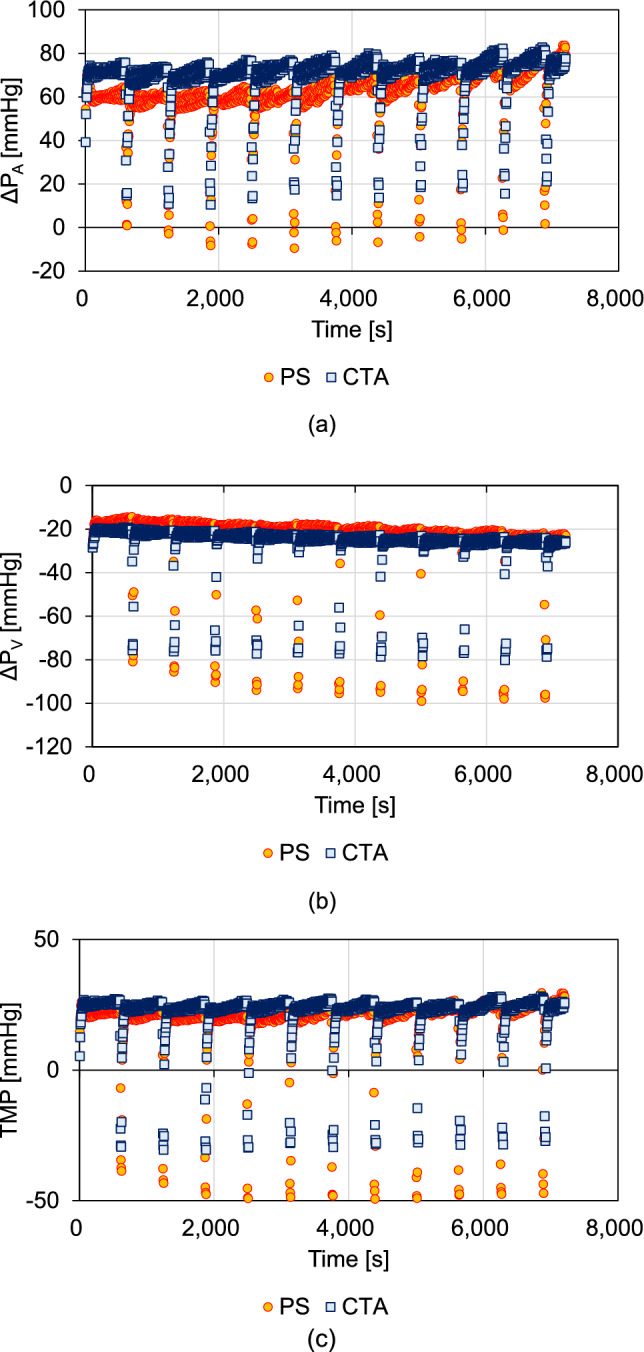
Changes in the pressure differences between the inlet portion (ΔP_A_) of hemodiafiltration membranes, outlet portion (ΔP_V_), transmembrane pressure (TMP), and Lp in the I-HDF mode. The pressures in the I-HDF mode are shown for **a** ΔP_A_, **b** ΔP_V_, and **c** TMP

Figure 4 shows the ΔP_A_, ΔP_V_, and TMP changes in the I-HDF mode. The ΔP_A_ was higher in the CTA than in the PS membrane (Fig. 4a). The absolute ΔP_V_ values during back filtration were higher in the PS than in the CTA membrane (Fig. 4b). Furthermore, both membranes decreased TMP during back filtration, and the absolute TMP values were lower in the CTA than in the PS (Fig. 4c).

**Fig. 5:**
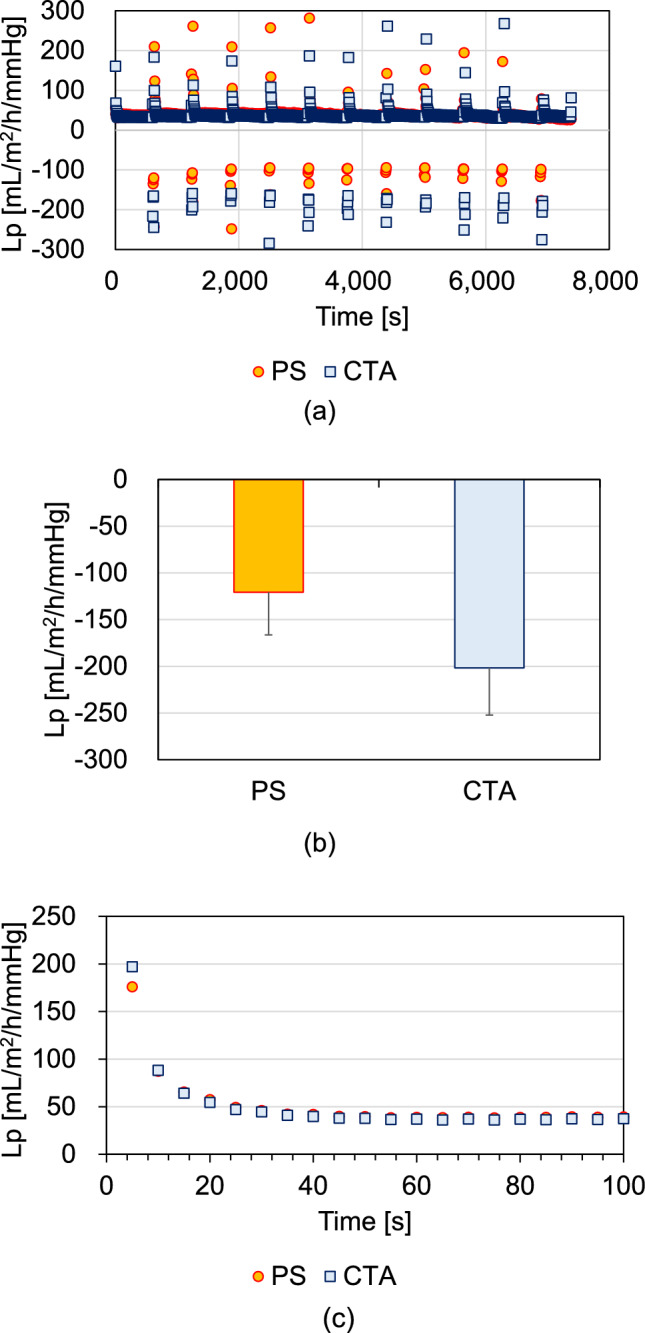
Lp changes in the I-HDF mode. **a** Positive and negative Lp values, **b** Lp values during back filtration in the I-HDF mode, and **c** mean positive Lp values for 100 s after back filtration. Data are described from the blood side to the dialysate side as a positive Lp and from the dialysate side to the blood side as a negative Lp

Figure 5 shows the Lp changes in I-HDF mode. The Lp values for the PS were mostly plotted from − 100 to − 150 mL m^−2^ h^−1^ mmHg^−1^ during the back filtrations, while those for the CTA were from − 150 to − 250 mL m^−2^ h^−1^ mmHg^−1^ (Fig. 5a). The Lp values during back filtrations were − 121 ± 46 and − 202 ± 50 mL m^−2^ h^−1^ mmHg^−1^ for PS and CTA, respectively (Fig. 5b). An increase in the absolute value of Lp during back filtration was observed. The mean positive Lp values (after 11 back filtrations) over 100 s after intermittent back filtrations were comparable for both membranes (Fig. 5c).

## Discussion

We conducted a comparative study using the continuously measured Lp as an index of the membrane surface conditions to clarify fouling formation in HD, pre-dilution online HDF, and I-HDF using two different membrane materials, PS and CTA. According to the manufacturer specifications for both membranes, the ultrafiltration rate (UFR) was 108 and 81 mL mmHg^−1^ h^−1^ in PS and CTA, respectively (Table 1).

The characteristics of each membrane were as follows: PS is a synthetic polymer membrane, and because of its hydrophobicity, polyvinylpyrrolidone (PVP), a hydrophilic agent, was added. PVP addition to the membranes helps control the pore size and improve biocompatibility [10, 11]. In contrast, conventional CTA is characterized by its low protein adsorption capacity; however, by creating an asymmetric structure, the interior of the hollow fibers becomes smoother, resulting in even less protein adhesion than conventional CTA membranes [12]. Namekawa et al. [13] evaluated the adsorption force of human serum albumin (HSA) on the inner surface of a PS membrane and the membrane surface smoothness using atomic force microscopy and found a correlation between the membrane roughness and HSA adsorption force. The smoother the inner surface, the easier it is for blood to flow near the membrane surface, and the greater the platelet activity suppression. Thus, the recently developed PS and CTA membranes have demonstrated excellent biocompatibility.

The results of this study showed that the Lp values in both membranes decreased immediately after the start of filtration in each mode (Fig. 2). In this experiment, a decrease in the Lp was observed within 20 s of the start of filtration, confirming primary fouling (first fouling) occurrence in both the PS and CTA membranes. Fouling is a process in which albumin, lipids, and globulin in the blood deposit membrane pores immediately after filtration. In this study, a high Q_F_ of 30 mL/min was set for each mode. In the I-HDF mode, hemoconcentration occurred at 6 mL/min until the first intermittent infusion because intermittent supplementation occurred at 10 min intervals. Consequently, the Lp in the I-HDF mode was slightly lower than that in the HD mode for both membranes (Fig. 2). In the pre-dilution online HDF, Lp temporarily increased after the first fouling; thereafter plateaued in the CTA and decreased in the PS membrane.

In pre-dilution online HDF, the initial rapid decrease in Lp was due to hemoconcentration in the HDF membranes occurring in the first 20 s (first fouling), after which diluted blood flowed into the HDF membranes and fouling occurred after 80 s (second fouling) (Fig. 2). The fiber lengths in the PS and CTA membranes were 288 and 283 mm, respectively (Table 1). It is estimated that blood flow from the arterial air trap to the center of HDF membranes takes approximately 20 s because the blood flow speed within the hollow fiber is 0.81 in HD and I-HDF and 1.64 cm/s in pre-dilution online HDF, respectively. The Lp results in Fig. 2 indicate that diluted blood appears after 20 s. The fouling steps can also be explained in terms of ΔP_A_, ΔP_V_, and TMP changes (Fig. 3). During the first fouling step (20 s after filtration), ΔP_A_ (Fig. 3a), ΔP_V_ (Fig. 3b), and TMP (Fig. 3c) increased owing to hemoconcentration in both membranes. Subsequently, ΔP_A_, ΔP_V_, and TMP decreased owing to hemodilution due to supplementation in the HDF membranes, marking the second fouling step process. The Lp was higher in pre-dilution online HDF than in HD and I-HDF because the protein concentration decreased owing to hemodilution by the supplemental fluid; moreover, the blood linear velocity was high, suppressing membrane fouling. As blood is a viscous fluid, shear stress is applied to the membrane when it flows through the lumen of a hollow fiber membrane. It is believed that the high blood linear velocity increases the shear stress exerted by the blood on the inner membrane surface, and, as a result, inhibits the formation of a concentration-polarized layer. As the Lp plateaued in the CTA and decreased in the PS membrane during the pre-dilution HDF mode, CTA is speculated to have low fouling properties during this mode.

In I-HDF, the Lp of both membranes changed in synchrony with intermittent infusion. The Lp during back filtration increased in both membranes (Fig. 2) because the initial Lp in I-HDF indicated that the HDF membrane was completely replaced with the bovine blood, whereas the Lp during back filtration was affected by hemodilution due to dialysis fluid supplementation. From the results of the ΔP_A_ changes, pressure differences from the blood side to the dialysate side (normal filtration) were higher in the CTA than in the PS membranes; this was also observed during the intermittent back filtrations (Fig. 4a). For the ΔP_V_, the absolute values were higher in the PS than in the CTA membranes during back filtration, indicating that high pressure is required for filtration from the dialysate side to the blood side in the PS membrane (Fig. 4b). Moreover, the TMP absolute values in the CTA were lower than those in the PS membranes (Fig. 4c). The absolute Lp values during back filtration were higher in the CTA membrane than in the PS membrane (Fig. 5b). However, the mean Lp over 100 s after back filtration was comparable for both membranes (Fig. 5c). These results suggest that while the flushing effect by back filtration is obtainable for CTA compared with PS, Lp recovery after back filtration is comparable for both membranes. Although UFR in PS is higher than that in CTA (Table 1), fouling occurred more readily in PS than in CTA membranes. Watanabe et al. [9] reported that an increase in the Q_F_ increases normal filtration. With an increasing Q_F_, a large clearance recovery may be expected through flushing via back filtration. In this study, ΔP_A_ absolute values were higher in CTA than in PS, indicating that normal filtration was obtained in CTA compared with PS. In addition, the ΔP_V_ absolute values during back filtration were lower in the CTA than in the PS membranes under high Q_F_ (30 mL/min) conditions. These results indicate that the flushing effect via back filtration is influenced by the membrane material and structure.

This study has some limitations. The number of studies in each mode was small, and albumin clearance was not measured. However, the hematocrit and protein concentrations were standardized to account for the variations due to the bovine blood influence. The Lp in the I-HDF mode of PS decreased during the first 20 s, then recovered over the next 80 s, probably owing to the bovine blood conditions. Furthermore, since lipids also affect the membrane pressure, errors may occur in an experimental system similar to an ex vivo study [14]. These effects should be considered. Although the new PS membrane had a higher UFR value than the CTA membrane, the negative Lp was clearly reversed in the I-HDF mode for both membranes, and the fouling characteristics in each mode could be understood.

## Conclusion

The first fouling was completed within 20 s of the start of ultrafiltration in each mode. An intermittent high-flushing effect owing to back filtration was observed in the I-HDF for both the PS and CTA membranes. The differences in fouling steps in each of the three modes—HD, pre-dilution online HDF, and I-HDF—can be identified by continuously measuring Lp values.

## Data Availability

All data analyzed during this study are included in this article.

## References

[1] Liyanage T, Ninomiya T, Jha V, Neal B, Patrice HM, Okpechi I, Zhao MH, Lv J, Garg AX, Knight J, Rodgers A, Gallagher M, Kotwal S, Cass A, Perkovic V. Worldwide access to treatment for end-stage kidney disease: a systematic review. Lancet. 2015;385:1975–82. 25777665 10.1016/S0140-6736(14)61601-9

[2] Nitta K, Abe M, Masakane I, Hanafusa N, Taniguchi M, Hasegawa T, Nakai S, Wada A, Hamano T, Hoshino J, Joki N, Goto S, Wakasugi M, Yamamoto K, Nakamoto H. Annual dialysis data report 2018, JSDT renal data registry: dialysis fluid quality, hemodialysis and hemodiafiltration, peritoneal dialysis, and diabetes. Ren Replace Ther. 2020;6:51.

[3] Kikuchi K, Hamano T, Wada A, Nakai S, Masakane I. Predilution online hemodiafiltration is associated with improved survival compared with hemodialysis. Kidney Int. 2019;95:929–38. 30782421 10.1016/j.kint.2018.10.036

[4] Peters SA, Bots ML, Canaud B, Davenport A, Grooteman MP, Kircelli F, Locatelli F, Maduell F, Morena M, Nubé MJ, Ok E, Torres F, Woodward M, Blankestijn PJ, HDF Pooling Project Investigators. Haemodiafiltration and mortality in end-stage kidney disease patients: a pooled individual participant data analysis from four randomized controlled trials. Nephrol Dial Transpl. 2016;31:978–84. 10.1093/ndt/gfv349 26492924

[5] Okada K, Michiwaki H, Tashiro M, Inoue T, Shima H, Minakuchi J, Kawashima S. Effects of Japanese-style online hemodiafiltration on survival and cardiovascular events. Ren Replace Ther. 2021;7:1–10.

[6] Mineshima M, Eguchi K. Development of intermittent infusion hemodiafiltration using UltraPure dialysis fluid with an automated dialysis machine. Blood Purif. 2013;35:55–8. 23466380 10.1159/000346371

[7] Mineshima M, Takahashi S, Tomo T, Kawanishi H, Kawaguchi H, Minakuchi J, Nakanishi T, Sato T, Nitta K, Tsuchiya K, Masakane I, Itami N. A clinical significance of intermittent infusion hemodiafiltration using backfiltration of UltraPure dialysis fluid compared to hemodialysis: a multicenter randomized controlled crossover trial. Blood Purif. 2019;48:368–81. 31311018 10.1159/000501511

[8] Mineshima M, Eguchi K, Shishido K, Takahashi S, Kubo T, Kawaguchi H, Shitomi K, Shibagaki K, Suga K, Nagao H, Takada M, Taoka M, Sato T. Clinical effectiveness of intermittent infusion hemodiafiltration using backfiltration of UltraPure dialysis fluid compared with predilution on-line hemodiafiltration. Contrib Nephrol. 2017;189:24–9. 27951546 10.1159/000450636

[9] Watanabe M, Kiguchi T, Yamashita AC. Novel substitution technique in intermittent infusion hemodiafiltration (I-HDF) therapy using back filtration as substitution. J Artif Organs. 2022;25:336–42. 35303204 10.1007/s10047-022-01321-8

[10] Streicher E, Schneider H. The development of a polysulfone membrane. A new perspective in dialysis? Contrib Nephrol. 1985;46:1–13. 4006470

[11] Hayama M, Yamamoto K, Kohori F, Uesaka T, Ueno Y, Sugaya H, Itagaki I, Sakai K. Nanoscopic behavior of polyvinylpyrrolidone particles on polysulfone/polyvinylpyrrolidone film. Biomaterials. 2004;25:1019–28. 14615167 10.1016/s0142-9612(03)00629-x

[12] Sunohara T, Masuda T. Fundamental characteristics of the newly developed ATA™ membrane dialyzer. Contrib Nephrol. 2017;189:215–21. 27951571 10.1159/000451044

[13] Namekawa K, Fukuda M, Matsuda M, Yagi Y, Yamamoto K, Sakai K. Nanotechnological characterization of human serum albumin adsorption on wet synthetic polymer dialysis membrane surfaces. ASAIO J. 2009;55:236–42. 19357497 10.1097/MAT.0b013e3181984229

[14] Kurihara Y, Ueki S, Kokubo K, Kobayashi Y, Ebine T, Murakami K, Ushiroda Y, Maruyama N, Tsukao H, Kobayashi K, Kobayashi H. Continuous hemofiltration model using porcine blood for comparing filter life. J Artif Organs. 2018;21:332–9. 30039456 10.1007/s10047-018-1060-3

